# Supplementary material for: Oviduct epithelial spheroids during in vitro culture of bovine embryos mitigate oxidative stress, improve blastocyst quality and change the embryonic transcriptome
Source: Biol Res. 2024 Oct 22;57:73. doi: 10.1186/s40659-024-00555-5 (PMC11494963; doi:10.1186/s40659-024-00555-5)
Supplement: Supplementary file 3 — Supplementary Material 3: Fig. 1. Representative images of blastocysts after nuclei staining. A-C, Blastocysts cultured under 5% oxygen (O2) alone (A) or with oviduct epithelial spheroids (OES) for 4 days (B) or 7 days (C). D-F, Blastocysts cultured under 20% oxygen (O2) alone (D) or with oviduct epithelial spheroids (OES) for 4 days (E) or 7 days (F). [file 40659_2024_555_MOESM3_ESM.docx]

**Oviduct epithelial spheroids during *in vitro* culture of bovine embryos mitigate oxidative stress, improve blastocyst quality and change the embryonic transcriptome**

Thanya Pranomphon^1,2^, Álvaro López-Valiñas^3^, Carmen Almiñana^3,4^, Coline Mahé^1^, Viviane Lopes Brair^5^, Rangsun Parnpai^2^, Pascal Mermillod^1*^, Stefan Bauersachs^3*§^, Marie Saint-Dizier^1*§^

*These authors contributed equally to this work.

^1^ INRAE, CNRS, Université de Tours, PRC, 37380, Nouzilly, France

^2^ Embryo Technology and Stem Cell Research Center, School of Biotechnology, Suranaree University of Technology, Nakhon Ratchasima, 30000, Thailand

^3^ Institute of Veterinary Anatomy, Vetsuisse Faculty, University of Zurich, 8315, Lindau (ZH), Switzerland

^4^ Department of Reproductive Endocrinology, University Hospital Zurich, 8091, Zurich, Switzerland

^5^ Universidade Federal Fluminense (UFF), Niterói, Rio de Janeiro, Brazil

^§^Correspondence

Marie Saint-Dizier, INRAE Centre Val de Loire, Nouzilly, 37380, France.

Tel.: +33 2 47 42 75 08, +33 2 47 36 70 14, E-mail: marie.saint-dizier@univ-tours.fr

Stefan Bauersachs, Vetsuisse Faculty, University of Zurich, 8315, Lindau (ZH), Switzerland.

Tel.: +41 44 635 89 18, E-Mail: stefan.bauersachs@uzh.ch

**Abstract**

**Background**. *In* *vitro* embryo production is increasingly used for genetic improvement in cattle but bypasses the oviduct environment and exposes the embryos to oxidative stress with deleterious effects on further development. Here we aimed to examine the effect of oviduct epithelial spheroids (OES) on embryo development and quality in terms of morphology and gene expression during two co-culture times (4 days: up to embryonic genome activation at 8-16 cell stage *vs*. 7 days: up to blastocyst stage) and under two oxygen levels (5% *vs*. 20%).

**Methods**. Bovine presumptive zygotes produced by *in vitro* fertilization (day 0) using in-vitro matured oocytes were cultured in droplets of synthetic oviductal fluid (SOF) medium with or without (controls) OES for 4 or 7 days under 5% or 20% oxygen (4 treated and 2 control groups). Cleavage rates were evaluated on day 2 and blastocyst rates on days 7-8. Expanded blastocysts on days 7-8 were evaluated for total cell numbers and gene expression analysis by RNA-sequencing.

**Results**. Under 20% oxygen, blastocyst rates and total cell numbers were significantly higher in the presence of OES for 4 and 7 days compared to controls (P<0.05), with no difference according to the co-culture time. Under 5% oxygen, the presence of OES did not affect blastocyst rates but increased the number of cells per blastocyst after 7 days of co-culture (P<0.05). Both oxygen level and OES co-culture had a significant impact on the embryonic transcriptome. The highest number of differentially expressed genes (DEGs) was identified after 7 days of co-culture under 20% oxygen. DEGs were involved in a wide range of functions, including lipid metabolism, membrane organization, response to external signals, early embryo development, and transport of small molecules among the most significantly impacted.

**Conclusion**. OES had beneficial effects on embryo development and quality under both 5% and 20% oxygen, mitigating oxidative stress. Stronger effects on embryo quality and transcriptome were obtained after 7 than 4 days of co-culture. This study shows the impact of OES on embryo development and reveals potential molecular targets of OES-embryo dialog involved in response to stress and early embryonic development.

**Keywords:** Oviduct, Spheroids, Oxidative stress, *In vitro* embryo production, Embryo, RNA-seq, Transcriptome, Cattle.

**Background**

The transfer of *in vitro* produced (IVP) embryos in cattle has drastically increased over the past 20 years and is now a major way to accelerate genetic progress for meat and milk production. According to the statistics of embryo production and transfer in domestic farm animals collected by the *International Embryo Transfer Society*, more than 1.5 million IVP bovine embryos were transferred worldwide in 2022 (1). However, the efficiency of IVP in farm animals is not optimal. The ability of cattle IVP embryos to survive after cryopreservation is much lower after *in vitro* than *in vivo* development (2, 3) and recipient cows receiving IVP embryos have on average 10 to 40% less chance to become pregnant than after insemination (2, 4, 5). A number of studies evidenced important differences between *in vitro* and *in vivo* derived bovine embryos in terms of morphology (6), ultrastructure (7, 8), lipid profiles (9), gene expression (10-12), microRNA cargos (13) and proteomic contents (14), highlighting the importance of the environment to which the oocyte and the embryo are exposed for future developmental competence.

Indeed, crucial events for the early bovine embryo take place in the oviduct: the first mitotic cell divisions, the switch from RNAs and proteins derived from the oocyte and sperm to those produced from the zygote, called embryonic genome activation (EGA), occurring at the 8-16 cell stage in cattle as in humans (15), and then the formation of first cell-cell junctions leading to morula compaction and cell lineage differentiation (4). Eventually, around 5 days after fertilization, the embryo enters the uterus and reaches the blastocyst stage for further development. Previous transcriptomic (12) and proteomic (14) data evidenced significant differences in the molecular dynamics during EGA between *in vivo* and *in vitro* derived embryos, suggesting that the oviduct environment has a significant impact on this key event. Furthermore, although the physiological oxygen tension in the oviduct lumen of most mammals ranges from 2 to 8% (16), a lot of laboratories culture embryos at atmospheric oxygen level (20%) for practical and budget reasons, causing oxidative stress and potential irreversible damage in developing embryos (17-20).

Co-culture of embryos with monolayers of oviduct epithelial cells (OEC) under 20% oxygen has been shown to increase blastocyst production and quality compared to controls without OEC in cattle (21-23). This positive effect may be due to a decrease in oxygen tension in the culture medium by OEC oxygen consumption, as well as direct positive effects of OEC secretions on embryo growth (22, 24-26) and gene expression (21, 23, 27), despite a partial dedifferentiation of OEC during culture (22). A previous study reported a higher blastocyst yield when embryos were co-cultured with OEC monolayer under 20% compared to 5% oxygen (23), suggesting that OEC need high oxygen level to better support embryo development. In order to overcome the dedifferentiation of OECs cultured in monolayers (22), we recently developed an *in vitro* model of oviduct epithelial spheroids (OES) with well-differentiated isthmic epithelial cells preserved during the 8 days of embryo co-culture (28). A positive effect of OES on blastocyst yield after 8 days of co-culture under 20% oxygen was evidenced (28). However, the optimal time and oxygen level for *in vitro* embryo production by OES co-culture has not been determined. We hypothesized that OES supplementation up to the EGA, i.e. the first 4 days of culture, would be necessary and sufficient to obtain a supportive effect on embryo development and quality. Furthermore, although there is evidence that the oviduct secretions alter the embryo transcriptome (29), most studies explored a limited number of candidate genes (21, 23, 27), calling for a bigger picture of the impact of OES on the embryonic transcriptome.

Therefore, the objectives of this study were to examine the effect of OES according to the co-culture time (4 vs. 7 days) and oxygen levels (5% vs. 20%) on embryo development and blastocyst quality in terms of morphology and transcriptome profile.

**Methods**

**Chemicals and Reagents**

All chemicals and reagents were purchased from Sigma-Merck (Saint Louis, MO, USA), unless otherwise stated.

Phosphate-Buffered Saline (PBS) (1X, Eurobio Scientific, France, CS1PBS01-01), 4% paraformaldehyde (Santa Cruz Biotechnology, SC-281692), Triton X-100 (9036-19-5), bovine serum albumin (BSA; A9647), Hoechst 33342 (B2261; 1 mg/mL), mineral oil (ORIGIO Denmark), miRNeasy Tissue/Cells Advanced Micro Kit (#217684, Qiagen Basel, Basel, Switzerland), SMARTer® Stranded Total RNA-Seq Kit v3 - Pico Input Mammalian (#634485, Takara Bio Europe SAS, Saint-Germain-en-Laye, France).

**Culture of bovine oviduct epithelial spheroids (OES)**

Oviductal cell isolation and culture of OES were conducted as described previously (28). Briefly, pairs of oviducts and ovaries obtained from post-pubertal cows were collected at a local slaughterhouse (Vendôme, France) and transported at 4°C to the laboratory. Pairs of oviducts at the peri-ovulatory phase of cycle (approximately day -2 to day +4 around ovulation time) were selected according to the morphology of the ovaries. After removal of blood vessels and connective tissue, the oviducts were cut at the ampullary-isthmic junction and only the isthmic parts were used. After a rapid dip in 70% ethanol and rinsing in 0.9% NaCl, the mucosa fragments were expelled from isthmic sections by squeezing with forceps into a 15-mL conical tube (Thermo Scientific) containing 10 mL of TCM199 (Gibco), vortexed for 1 min to produce smaller fragments, then incubated at 38.8°C for 10 min for cell sedimentation. Following the elimination of the supernatant containing cell debris and blood red cells, the pellet was resuspended in 10 mL of M199, and the vortex-sedimentation process was repeated. Finally, the pellet was diluted ten times in the culture medium and 50 µL of the resulting mixture was added to 450 µL of M199, to reach a 100-fold final dilution. The isthmic mucosa fragments were cultured for 72 h in four-well culture plates (Thermoscientific, Denmark) at 38.8°C in a humidified atmosphere containing 5% CO_2_ in air. After 72 h, a cavity appeared within the mucosa fragments, forming floating spheroids of various sizes and shapes, with the apical side of the epithelial cells oriented outward. The spheroids between 100 and 200 µm in diameter, homogeneous in shape and size and exhibiting a cavity and ciliary beating outward, referred as “OES”, were selected using an ocular micrometer under a stereomicroscope (see the morphology and movements of OES in Suppl. video 1). The OES were previously characterized for morphology, cell number, viability and oviduct epithelial cell markers (28). The OES were cultured in M199 medium at a density of 200 to 400 OES/mL for 2 days and then, transferred to culture droplet for 24 h before co-culture with presumptive zygotes.

***In vitro* oocyte maturation (IVM), fertilization (IVF) and embryo culture**

IVM, IVF and *in vitro* culture were performed as previously described (22) with slight modifications. Bovine ovaries (mix of breeds) were collected at a local slaughterhouse (Vendôme, France) and kept in 0.9% NaCl solution at 31-32°C for 45 min up to the laboratory. Cumulus oocyte complexes (COCs) were aspirated from 3-6 mm follicles using an 18½-gauge needle. COCs surrounded by three or more layers of compact cumulus and homogeneous cytoplasm were selected, washed twice in washing medium then once in 1 mL of maturation medium. Groups of 40-50 COCs were cultured in 500 μL of maturation medium for 22-23 h at 38.8°C in a humidified atmosphere of 5% CO_2_ in air in a 4-well plate (Thermo Scientific™ REF179830, Denmark). The COCs were washed once in 1 mL of fertilization medium and transferred in groups of 40-50 into 4-well dishes containing 250 µL of fertilization medium per well. A pool of frozen semen from two Holstein bulls of proven fertility were used for all IVF (0.25-mL straw). Straws were submerged in a 35°C water-bath for 30 sec then motile spermatozoa were obtained by centrifuging frozen-thawed semen on a Percoll density gradient (45/90%) at 700g for 20 min. Spermatozoa collected at the bottom of the 90% fraction were centrifuged in 5 mL of STL medium at 100g for 10 min, before counting spermatozoa in the pellet using a Thoma cell. Sperm concentration was adjusted to 4x10^6^ spermatozoa/mL with fertilization medium then 250 μL of this suspension were added to 40-50 COCs to obtain a final concentration of 2x10^6^ spermatozoa/mL in a final volume of 500 μL of fertilization medium per well. Spermatozoa and COCs were co-incubated at 38.8°C in a humidified atmosphere containing 5% CO_2_ in air (IVF, day 0). After 18 h of co-incubation, the presumptive zygotes (day-1 embryos) were vortexed at moderate speed for 2 min in 2 mL of washing medium to eliminate cumulus cells, washed twice in washing medium then once in SOF medium supplemented with 5% FBS before being transferred in 25 µL droplets of the same medium overlaid with mineral oil at 38.8°C. Groups of 25 presumptive zygotes were randomly allocated to one of the following experimental group: co-culture with 25 OES in a humidified atmosphere with 5% CO_2_, 5% O_2_ and 90% N_2_ (low O_2_ condition) up to day 5 i.e., for 4 days (**4dOES_5**), or up to day 8 i.e., for 7 days (**7dOES_5**) of culture, or in a humidified atmosphere with 5% CO_2_ in air (high O_2_ condition) up to day 5 (**4dOES_20**) or day 8 (**7dOES_20**) (Fig. 1). In both conditions, one group of 25 zygotes was cultured without OES as controls (**C_5**; **C_20**). In the 4dOES groups, OES were removed from the culture droplet with minimal volume of medium (<5 µL) using a glass pipette. The cleavage rates (number of cleaved embryos reported to the total number of presumptive zygotes on day 1) were evaluated on day 2 and blastocyst rates (number of blastocysts reported to the total number of presumptive zygotes on day 1) on days 6, 7, and 8 using an inverted microscope (Olympus IX70, Japan).

**Assessment of blastocyst cell numbers**

On days 7 and 8, subgroups of expanded blastocysts (around 150 µm in diameter) were fixed and permeabilized in 500 µL PBS with 4% paraformaldehyde (PFA), 1% BSA and 0.25% Triton X-100 for 30 min at 37°C. After three washing in PBS + 1% BSA (PBS-BSA), embryos were stained in 1 µg/mL Hoechst 33342 for 1 h at room temperature under agitation in dark, rinsed and mounted on a glass slide. Embryos were observed at 200x magnification using a confocal microscope (Zeiss LSM 700, Carl Zeiss, Oberkochen, Germany) and Z-stack images (2 to 7 per blastocyst) were acquired. The total number of nuclei per embryo was counted automatically using the Stardist plugin of the ImageJ software (version 1.54f). The counting of all nuclei and absence of double counting were checked visually and corrected manually when necessary.

**RNA-sequencing analysis**

Expanded blastocysts on days 7-8 of the six culture conditions (C_5, 4dOES_5, 7dOES_5, C_20, 4dOES_20 and 7dOES_20) were washed in washing medium, collected with minimum volume of medium in 1.5 mL Ultra High Recovery Microcentrifuge Tube (STARLAB, E1415-2600, USA), immediately snap-frozen in liquid nitrogen and stored at –80°C until used for RNA extraction. Total RNA was isolated from pools of 8-12 blastocysts using the miRNeasy Tissue/Cells Advanced Micro Kit (#217684, Qiagen Basel, Basel, Switzerland) following the manufacturers’ recommendations. RNA concentration and quality were measured on an Agilent 2100 Bioanalyzer (Agilent Technologies Schweiz AG, Basel, Switzerland) using the Agilent RNA 6000 Pico assay. The Agilent 2100 Bioanalyzer RNA integrity number (RIN) ranged from 9.3 to 10 (median= 9.9).

RNA-seq library preparation was performed starting from 2 ng total RNA using the SMARTer® Stranded Total RNA-Seq Kit v3 - Pico Input Mammalian (#634485, Takara Bio Europe SAS, Saint-Germain-en-Laye, France). A pool of 36 libraries was run on one lane of an Illumina NovaSeq X plus 10B flow cell (Functional Genomic Center Zurich, https://fgcz.ch/). Paired-end 150 bp sequencing was performed and revealed between 21 and 36 million raw reads per library.

The Galaxy Europe server (https://usegalaxy.eu/ (36)) was used to analyze the obtained sequence reads (FastQ files). Sequencing reads were processed using Trim Galore! (Galaxy version 0.6.7+galaxy0) with the parameters: remove 14 bp from the 3' end of read 1; remove 6 bp from the 3' end of read 2; trim low-quality ends from reads in addition to adapter removal (phred quality score threshold = 30); overlap with adapter sequence required to trim a sequence: 1; maximum allowed error rate: 0.1; discard reads that became shorter than 30 nt; remove 9 bp from the 5' end of read 1; remove 16 bp from the 5' end of read 2. Trimmed reads were mapped to the current bovine genome reference assembly (bosTau 9, ARS-UCD2.0) with HISAT2 (Galaxy version 2.2.1+galaxy1). Reads mapped to annotated features of the bovine genome were counted with the tool featureCounts (Galaxy Version 2.0.3+galaxy2). The latest NCBI GFF3 genome annotation file was used (GCF_002263795.3_ARS-UCD2.0_genomic.gtf). The obtained read count table was filtered based on counts per million (cpm) cut- off 1.65 (corresponds to approx. 20 read counts) in at least 5 samples to remove reads with negligible read counts. Read count data were analyzed in R software (https://www.r-project.org) using the BioConductor package edgeR (37). False discovery rate (FDR) was calculated to perform correction for multiple testing. Since the degree of correction of nominal P-values depends on the number of potentially differentially abundant genes, different FDR cut-offs were used for the various group comparisons. The problem of the too strict P-value correction in case of very low numbers of DEGs has been described (38).

Hierarchical clustering was performed to identify clusters of differentially expressed genes (DEGs) with similar expression profiles across experimental groups (Multiple Experiment Viewer, MeV v.4.8.1, <https://sourceforge.net/projects/mev-tm4/>) (39). Functional annotation analysis for lists of DEGs was performed using Metascape ([www.metascape.org](http://www.metascape.org)) (40). The webtool ToppCluster (<https://toppcluster.cchmc.org/>) (41) was used to generate a network of overrepresented functional terms obtained for DEGs.

**Validation of selected DEGs by quantitative RT-PCR**

The selection of genes for validation of differential expression was based on the top overrepresented and relevant functional categories and fold change. Primers for the selected genes were designed using Primer-BAST tool (<https://www.ncbi.nlm.nih.gov/tools/primer-blast/>) (Supplementary table S1). The cDNA synthesis and amplification was performed using SuperScript™ IV Single Cell/Low-Input cDNA PreAmp Kit (#2852631, Thermo Fisher Scientific, Reinach, Switzerland) according to the manufacturer’s instructions, using 2 ng total RNA as input (same RNA samples as used for RNA-seq). Amplification products were purified with RNAClean XP (A63987, Beckman Coulter International S.A., Nyon, Switzerland). Elution from the beads was performed in 30 µl and 20 μl TE buffer were added to a final volume of 50 µl. Real-time PCR was performed in a LightCycler® 96 Real-Time PCR System (Roche Diagnostics (Schweiz) AG, Rotkreuz, Switzerland) in 20 µl reactions, consisting of 10 μl of KAPA SYBR FAST LC480 (KK4610, Merck & Cie, Buchs, Switzerland), 1.2 µl of 5 mM each forward and reverse primers (0.3 μM final), 7.8 μl RNase-free water, and 1 μl of each cDNA sample, using Hard-Shell® 96-Well PCR Plates, low profile, clear/white, barcoded (#HSR9905, Bio-Rad Laboratories AG, Cressier, Switzerland). The thermal cycle conditions were set as follows: 95°C for 180 s; 40 cycles of denaturation (95°C for 20 s), annealing (58 to 64°C for 15 s), and extension (72°C for 15 s); melting (95°C, 65°C and 97°C for 10, 60 and 1 s, respectively); and cooling (37°C for 10 s). Obtained quantification cycle (Cq) values were analyzed using the 2(-Delta Delta C(T)) method (42). Normalization was based on the averaged Cq values of the reference genes: *ACTB* (NM_173979.3), *GAPDH* (NM_001034034.2), *H3-3A* (NM_001014389.2), and *UBB* (NM_174133.2).

**Statistical analysis**

Statistical analysis was performed using the GraphPad Prism software (version 8.1.1) and the Rstudio (R version 4.3.3) (43). The normality of data and homogeneity of variances were analyzed using the Shapiro-Wilk and Levene tests, respectively. The effect of OES co-culture on the rates of cleavage, blastocysts and mean cell number per expanded blastocyst was analyzed using one-way ANOVA followed when appropriate by Tukey's post-tests. The effect of oxygen tension (5% vs. 20%) on cleavage, blastocyst rates and mean cell number per expanded blastocyst was analyzed using unpaired t tests with Welch’s correction for unequal variances. The qRT-PCR data were compared between groups using t-tests. A P-value < 0.05 was considered as significant. All data are presented as means ± SEM.

**Results**

**Effect of OES and time of co-culture on blastocyst yield and cell number**

Under 5% O_2_, the culture of embryos with OES did not change cleavage or blastocyst rates on days 7-8 compared to controls without OES, regardless the time of co-culture (Fig. 2A and Table S2). The mean cell number per expanded blastocyst co-cultured with OES for 7 days was higher than controls (102.6 ± 8.4 vs. 137.6 ± 10.8; *P*<0.05; Fig. 2A and Table S2; see supplementary Fig. 1 for representative pictures of blastocysts after nuclei staining).

Under 20% O_2_, cleavage rates did not change among culture conditions. The co-culture with OES increased the blastocyst rates on days 7 and 8 (*P*<0.01) versus control, but without differences between 4 and 7 days of co-culture (Fig. 2B and Table S2). Both 4 and 7 days of co-culture with OES increased the mean cell number per blastocyst compared to controls (*P*<0.0001). Moreover, 7 days of co-culture with OES tended to have a higher effect on blastocyst cell number than 4 days of co-culture (112.7 ± 7.8 vs. 138.1 ± 10.5; *P*=0.07; Fig. 2B and Table S2).

**Effect of oxygen level on blastocyst yield and cell number**

When control, OES for 4 days or OES for 7 days experimental groups were compared under different oxygen level (5 *vs*. 20%), statistical differences were only found for controls groups (Fig. 2). Both the blastocyst rate on day 8 and cell number per expanded blastocyst were lower under 20% compared to 5% oxygen in control groups (*P*<0.05; Fig. 2 and Table S3).

**Effect of co-culture with OES, co-culture time and oxygen level on the blastocyst transcriptome**

***Comparisons of OES-embryo co-culture versus controls***

The numbers of DEGs for the comparisons 4dOES *vs*. control and 7dOES *vs*. control at both 5% and 20% O_2_ are shown in Table 1 (top 4 lines). Higher numbers of DEGs were identified for the comparisons between 7dOES and controls (911 and 1282 DEGs for 5% and 20% O_2_, respectively; FDR 1%; Table 1 and Supplementary data 1, Sheet 1). The comparison 4dOES *vs*. control revealed similar numbers of DEGs regardless O_2_ level (568 and 559 DEGs for 5% and 20% O_2_, respectively; FDR 1%). The overlaps of DEGs between these four comparisons are shown in a Venn diagram in Fig. 3A. The overlaps of DEGs between 4dOES vs. control and 7dOES *vs*. control were 69% at 5% O_2_ and 65% at 20% O_2_. In addition, the overlaps of DEGs in the 5% vs. 20% O_2_ comparison were 31% for 4dOES *vs*. control and 56% for 7dOES *vs*. control.

Hierarchical clustering of the DEGs obtained for the four comparisons of co-culture vs. control groups revealed more pronounced differences between controls and OES co-culture groups when incubated at 20% O_2_ (Fig. 4). Furthermore, for a given oxygen level, gene expression differences were more distinct after 7 than 4 days of co-culture.

***Comparisons of 4 versus 7 days of co-culture***

The comparison between 4 and 7 days of co-culture revealed a higher number of DEGs at 20% compared to 5% O_2_ level (Table 1; Supplementary data 1, Sheet 2). At 5% O_2_, only two DEGs could be identified whereas 292 (FDR 10%) were obtained for 20% O_2_ level. The two DEGs identified for 5% O_2_ were also significant for 20% O_2_ level (Fig. 3B). Hierarchical cluster analysis of DEGs confirmed that differences between 4 and 7 days of co-culture were only present at 20% O_2_ level (Fig. 5).

***Comparisons of 5% versus 20% O_2_***

Gene expression in embryos was also compared between 5% and 20% O_2_ level. This was performed for the controls, 4dOES, and 7dOES (Table 1; Supplementary data 1, Sheet 3 and Fig. 6). A higher number of DEGs was obtained for controls and 4dOES comparisons (767 and 850 at FDR 5%, respectively). The comparison of 5% *vs*. 20% O_2_ for the 7dOES group resulted in the lowest number of DEGs (127 at FDR 5%). The three lists of DEGs were compared in a Venn diagram showing a relatively low overlap between these comparisons (Fig. 3C). The hierarchical cluster analysis for all DEGs illustrated in Fig. 6 clearly showed that many gene expression changes induced by increased O_2_ level in the control groups were also present in the 4dOES group. In contrast, less effects of increased O_2_ level were observed in the 7dOES 20% O_2_ group compared to the 7dOES 5% O_2_ group (Fig. 6).

**Functional categories overrepresentation analysis**

***Analysis of DEGs obtained as effect of OES presence***

Metascape functional enrichment analysis was performed for the DEG lists of the four comparisons between OES co-culture and control groups to characterize the impact of the presence of OES on embryonic gene expression. Fig. 7 shows a heatmap of the top 100 most significant functional terms of the four comparisons (additional details in Supplementary data 2, Sheet 1). Many functional terms, including “metabolism of lipids”, “response to extracellular stimulus”, “response to oxygen levels”, “cell-cell junction”, “import into cell” and “transport of small molecules” were found with similar significance for all four comparisons, i.e. impacted by OES co-cultured regardless of the oxygen level and time of co-culture. Most of the terms showed higher significance for the DEGs of 7dOES *vs*. control under 20% O_2_. The functional categories “mitochondrial transport”, “amino acid metabolic process” were only enriched for the comparisons under 20% O_2_ (4dOES–C 20% and 7dOES–C 20%).

To provide a graphical illustration of shared and specific overrepresented functional categories for the four comparisons, a network shown on Fig. 8 was generated using ToppCluster. The network evidenced that DEGs of the 7dOES–C 20% comparison were particularly overrepresented for “regulation of phosphorylation”, “vesicle membrane”, “regulation of apoptotic signalling pathways”, “transport of small molecules”, “metabolism of lipids”, and “endocytosis” among the most significant.

***Analysis of the DEGs obtained as effect of co-culture time***

The functional term enrichment analysis for the DEGs of the 4dOES *vs*. 7dOES comparisons revealed overrepresented terms for 4dOES *vs*. 7dOES under 20% O_2_ related to “import into cells”, “vesicle-mediated transport to the plasma membrane”, “chordate embryonic development”, and “focal adhesion” among the most significant (Supplementary data 2, Sheet 2).

***Analysis of the DEGs obtained as effect of oxygen level***

A heatmap of the top 100 most significant functional terms of the Metascape functional enrichment analysis of the DEGs between 5% and 20% O_2_ for the three group comparisons of blastocysts co-cultured with OES for 4 days, 7 days, and controls is shown in Fig. 9. Biological functions/pathways enriched in all groups were “Wnt signaling”, “regulation of cytoskeleton organization”, “protein kinase activity” and “tissue morphogenesis”, among others. Some functional categories were specifically enriched for embryos cultured alone, e.g., “cell junction organization” and “regulation of protein stability”, and “nuclear receptor activity”. Many functional terms were only found for blastocysts co-cultured with OES for 4 days, including “carbohydrate metabolite process”, “structural constituent of chromatin”, and “signaling by interleukins”.

**Validation of selected DEGs by real-time quantitative RT-PCR**

Differentially expressed genes were selected for quantitative real-time RT-PCR based on functional category overrepresentation analysis and the observed fold changes between group comparisons. For the nine genes listed in Table 2, results from RNA-seq were confirmed. For some comparisons, *P* values from *t*-test did not reach significance (*P* < 0.05), but the fold change was confirmed, and a tendency observed (*P* = 0.1).

**4. Discussion**

**4.1 OES improved embryo development and quality**

The improvement of both embryo development and quality in terms of total cell numbers under high oxygen level due to co-culture with OES is consistent with previous studies that used OEC monolayers under 20% oxygen, in which blastocyst yields were improved on days 7-8 compared to embryos cultured alone (21, 22, 28). Although we did not compare oxygen levels in the culture medium with or without OES, it is probable that this beneficial effect was partly due to oxygen consumption by OES in the culture medium, leading to an oxygen tension very close to the 2-8% in the oviduct lumen (16). Furthermore, the oviduct epithelium expresses antioxidant enzymes (glutathione peroxidase, superoxide dismutase and catalase) (22, 44) and antioxidant activities were measured in the oviduct fluid (44). There is evidence that the presence of OEC or the supplementation of OEC-derived extracellular vesicles (EVs) in the culture medium during embryo culture increase the expression of genes for enzymes involved in ROS scavenging, like glutathione peroxidase 1 (GPX1) (21) and superoxide dismutase 1 (SOD1) (47) in IVP embryos with concomitant decrease in embryonic ROS levels (47). In contrast for co-culture with OES, only expression of *GPX1* was slightly increased in embryos, while *GPX2* and *GPX3* were decreased. In control embryos, *GPX2* was increased under 20% O_2_. The expression of *SOD1* was also found as decreased for 7dOES under 5% O_2_ as well as the expression of another antioxidation-related gene, ubiquitin C-terminal hydrolase L1 (*UCHL1*) (48). Furthermore, expression of NAD(P)H quinone dehydrogenase 1 (*NQO1*), a gene with a known role in response to oxidative stress (49), was decreased by 7 days of co-culture (5% and 20% O_2_). This suggests a different effect of OES compared to OEC monolayers. Additionally, it could be speculated that under high O_2_ level, OES are under optimal conditions to support embryo development through passive oxygen consumption, active antioxidant secretions as well as transfer of EV-mRNAs and proteins helping the embryos to mitigate the effect of oxidative stress.

In this study, total cell number per blastocyst was used as the main criterion determining morphological quality of the embryos since this parameter has been positively correlated with pregnancy rates after embryo transfer in cattle (50, 51). Overall, co-culture with OES under both O_2_ levels significantly improved blastocyst growth, resulting in higher total cell numbers compared with embryos cultured alone. This is in line with previous reports in which the supplementation of the culture medium with low amounts of oviduct fluid (52) or oviduct fluid-derived EVs (25) supported blastocyst rate and blastocyst cell number. The beneficial effect of OES could be the result of an inhibition of cell apoptosis in blastomeres, as previously shown for OEC monolayers in pigs (47) and cattle (21), as well as a stimulation of cell mitosis. The embryonic transcriptome changes induced by OES co-culture suggest an inhibition of apoptosis, as indicated by overrepresentation of functional categories related to regulation of apoptosis. For example, co-culture with OES resulted in downregulation of caspase 8 (*CASP8*) mRNA, known to initiate the apoptosis signal in cells, and upregulation of caveolin 1 (*CAV1*) and nuclear protein 1, transcriptional regulator (*NUPR1*) mRNAs, both genes described with anti-apoptotic functions (55, 56). Also, genes described in the context of embryo development were found as upregulated by OES co-culture, e.g., transcription factor AP-2 alpha (*TFAP2A*) described as a trophectoderm lineage-specific gene in bovine embryos (57). On the other hand, genes with a positive effect on cell proliferation, such as cyclin dependent kinase 3 (*CDK3*) and estrogen receptor 1 (*ESR1*), were rather downregulated in co-cultured blastocyst compared to controls at 20% O_2_.

Overall, our results showed that the presence of OES during embryo culture had a significant impact on the blastocyst transcriptome. This effect on the embryonic transcriptome increased with co-culture time and O_2_ level. However, when considering the effect of OES, most of the enriched functional terms were shared among the four comparisons between co-cultured embryos and controls (i.e. very few terms were specific to one comparison), with higher significance after 7 than 4 days at both O_2_ levels. This indicates that longer co-culture time did not change the activated pathways but mostly the magnitude of gene expression changes.

Among the most significant functional terms enriched for the DEGs of all four comparisons were “transport of small molecules” and “metabolism of lipids”. For the transport, the ATP binding cassette family was represented by several subfamily members. ATP binding cassette subfamily G member 5 (*ABCG5*) and *ABCG8* expression was increased in co-cultured embryos, which may indicate higher antioxidant capacity in the context of a report showing positive correlation of expression with antioxidant capacity in rat liver cells (58). Looking at the many members of the solute carrier (SLC) family, the majority of the corresponding genes were found with increased expression in co-cultured embryos. Particularly, transport (uptake) of amino acids, which was overrepresented for the upregulated SLC family transporters, has been shown to improve embryo development (59, 60) and to protect from oxidative stress (61).

Metabolism of lipids seems to be particularly important for embryo development *in vitro* since bovine IVP embryos have high lipid accumulation and different phospholipid profiles (9) compared to their *in vivo* counterparts (62). A closer look at the DEGs assigned to “metabolism of lipids” revealed that the most significantly overrepresented terms were related to fatty acid, arachidonic acid, sphingolipid, cholesterol metabolism, and steroid biosynthesis. Interestingly, prostaglandin E synthase (*PTGES*) was increased in co-cultured embryos compared to controls, suggesting more advanced development of co-cultured embryos, in line with the results of Boruszewska *et al.* (65) and Saint-Dizier *et al.* (66). Hydroxy-delta-5-steroid dehydrogenase, 3 beta- and steroid delta-isomerase 1 (*HSD3B1*), the gene with highest upregulation in all co-cultured embryo groups compared to controls (fold-changes of 3 to 3.7 among the four comparisons), is a key gene in steroid hormone production (67) and has been previously detected in bovine pre-hatching blastocysts (68). The increased expression in co-cultured embryos further supports advanced embryo development. Related to the above-mentioned lipid accumulation in IVP embryos, increased expression of genes related to sphingolipid metabolism, such as sphingomyelin phosphodiesterase 1 (*SMPD1*), could be of interest.

**4.2 OES for 4 days are sufficient to improve embryo development but prolonging to 7 days increase the beneficial effects**

In cattle, at the beginning of cleavage, the embryo employs maternal mRNAs and proteins and is almost transcriptionally inactive, with only minor transcription activity up to the 4-cell stage (72). However, at the 8-16-cell stage in cattle, the embryonic genome is activated (major EGA) to initiate transcription and translation so that the embryo produces its own mRNAs and proteins while maternal transcripts and proteins decrease and disappear (72-74). Although the quality of the oocyte is important for blastocyst yield, there is evidence that the quality of the environment to which the embryo is exposed after fertilization has a great impact on EGA and RNA profiles in the resulting blastocysts (3, 5, 12, 21, 75, 76). Prior research from our group showed that the presence of OEC monolayers for the first 4 days of *in vitro* development was sufficient to improve blastocyst rates in cattle (21), yet with OEC partial dedifferentiation during co-culture (22). Here we validated the hypothesis that OES up to the EGA is enough to support both embryo development and quality. This was particularly obvious under 20% oxygen as there was no significant difference in blastocyst yield and cell numbers between the 4dOES_20 and 7d0ES_20. In the same line, the number of DEGs obtained when considering the effect of co-culture time was very low at 5% O_2_ and only 292 at 20% O_2_ (FDR 10%), showing that OES presence beyond the 16-cell stage had little additional impact on the embryonic transcriptome. However, under 5% O_2_, 7 days of co-culture had a beneficial effect of OES on blastocyst cell numbers. Therefore, although 4 days of co-culture correspond to the physiological exposure of embryos to the oviduct environment *in vivo*, it appears more beneficial and practical to keep OES with embryos for 7 days. Furthermore, the functional categories related to the DEGs between 7dOES and 4dOES at 20% O_2_ confirmed the additional supportive effect of 7 days co-culture on embryo development.

As for the co-culture comparison with controls, *CASP8* was downregulated and *CAV1* was upregulated in 7dOES compared to 4dOES at 20% O_2_, further suggesting that longer co-culture time has a stronger anti-apoptotic effect. Among the genes with higher levels in 7dOES at 20% O_2_, was zona pellucida glycoprotein 3 (*ZP3*), which has been reported as important for EGA and blastocyst formation in the mouse (80). Furthermore, a study of gene expression in goat embryos revealed WEE2 oocyte meiosis inhibiting kinase (*WEE2*) and growth differentiation factor 9 (*GDF9*) as increased in blastocysts cultured alone under 5% O_2_ compared to 20% O_2_ (81). Both *WEE2* and *GDF9* mRNAs were increased in 7dOES compared to 4dOES under 20% O_2,_ supporting the hypothesis that co-culture with OES is decreasing O_2_ tension in the embryo culture medium. Additional evidence for involvement of genes with increased expression in 7dOES at 20% O_2_ in embryo development is indicated by the three genes, KH domain containing 3 like, subcortical maternal complex member (*KHDC3L*), *WEE2*, and *ZP3*, which belong to 16 genes for which mutations were found as causative for oocyte maturation arrest, fertilization failure, embryonic arrest, and preimplantation embryonic lethality in humans (82). Moreover, for NOBOX oogenesis homeobox (*NOBOX*) expression in mouse embryos, a positive correlation with developmental competence has been found (83).

**4.3 OES differentially modulate the effect of oxygen tension on the blastocyst transcriptome**

In this study, the 20% O_2_ condition was considered as oxidative stress and 5% O_2_ as physiological for embryos. Considering this, the higher oxygen level was used to test the protective effect of OES toward embryos put under oxidative stress. Blastocyst rate and cell number were decreased in the control embryo group at 20% compared to 5% O_2_, which were rescued by only 4 days of co-culture with OES. Even though the 4dOES embryos were without OES for the last three days of culture, the overlap of DEGs between 20% and 5% O_2_ was very low between 4dOES and controls (84 DEGs; Fig. 3C). This suggests a long-lasting positive effect of 4dOES co-culture on the embryos. Furthermore, the low number of DEGs between 20% and 5% O_2_ for 7dOES (127 with FDR 5%), suggests that the permanent presence of OES is lowering O_2_ tension in the culture medium. This is further supported by the finding that functional terms related to oxidative stress were mainly overrepresented for controls 20% vs. 5% O_2_. However, only a few genes assigned to these categories were found as DEG for 4dOES and for controls. Only early growth response 1 (*EGR1*) was significant for all three groups with lower expression at 20% O_2_. This transcription factor is involved in regulation of hypoxia inducible factor 1 subunit alpha (*HIF1A*) during hypoxia (84).

The DEGs 20% *vs*. 5% O_2_ of the control group were specifically enriched for some categories related to nuclear receptors and transcription regulators. The gene msh homeobox 1 (*MSX1*) was only significantly increased (adj. *P* value <0.001) at 20% O_2_ in the control group. A study in bovine early embryos showed high expression in oocytes with a sharp decline from zygote to morula and blastocyst stages (85). In this context, the higher *MSX1* levels in the 20% O_2_ control group suggest delayed EGA or abnormally high expression in this embryo group. Another interesting group of transcription factors affected by oxygen levels seems the identified members of the nuclear receptor subfamily, since nuclear receptor subfamily 5 group A member 2 (*NR5A2*) has been described as a pioneer factor in EGA (86). In contrast, the transcription factors glial cells missing transcription factor 1 (*GCM1*) and hepatocyte nuclear factor 4 alpha (*HNF4A*), which were decreased in the 20% O_2_ control group, have been associated with trophoblast (87) and ICM development (88), respectively. Looking at categories related to oxidative stress, the GO category “cellular response to oxidative stress” was only significantly overrepresented for the DEGs of controls 20% vs. 5% O_2_. Other more general terms related to response to stress were also significant for 4dOES 20% vs. 5% O_2_, e.g., “Cellular responses to stress”. Some of the corresponding genes were found as significant for both comparisons. One of the genes which were only significantly upregulated for 4dOES 20% vs. 5% O_2_ was interleukin 6 (*IL6*). Recent studies showed that supplementation with IL6 during IVP increased bovine conceptus elongation and developmental competence (89). Another study of the same group revealed IL6 as an embryokine in bovine embryos that specifically increases ICM cell numbers and increased cleavage and day 8 blastocyst formation in embryos cultured individually (90). The expression profile of *IL6* over all samples shows that expression is increased in both control groups and in 4dOES 20%, suggesting that if IL6 is needed for proper embryo development, it might be provided by OES in co-culture as one of the mechanisms supporting embryo development. However, this needs to be confirmed in an additional study.

**Conclusion**

In conclusion, this study revealed for the first time the considerable impact of OES on embryo development, blastocyst quality, and gene expression under both 5% and 20% oxygen. Although 4 days of co-culture were enough to see beneficial effects of OES, maximal beneficial effect was seen after 7 days. Our results clearly show differential effects of OES on the embryonic transcriptome depending on oxygen tension and co-culture time, and pointing to a wide range of functions, including “transport of small molecules”, “lipid metabolism”, “intracellular transport and cell-cell junctions nucleosome”, “signaling by NOTCH”, and “ESR-mediated signaling” among the most significant functions modified by OES. Altogether, the study shows a clear modulation of the embryo-OES dialog according to the environment.

**Abbreviations**

IMF: isthmic mucosa fragments; OES: bovine oviduct epithelial spheroids; COCs: cumulus oocyte complexes; IVM: *in vitro* oocyte maturation; IVF: *in vitro* fertilization; SOF: the synthetic oviductal fluid medium; RNA-seq: RNA-sequencing; 4dOES: 4 days co-culture with OES; 7dOES: 7 days co-culture with OES; FDR: false discovery rate; DEGs: differentially expressed genes; 4dOES_5: 4 days co-culture with OES at 5% O_2_; 7dOES_5: 7 days co-culture with OES at 5% O_2_; C_5: 7 days culture with medium alone without OES at 5% O_2_; 4dOES_20: 4 days co-culture with OES at 20% O_2_; 7dOES_20: 7 days co-culture with OES at 20% O_2_; C_20: 7 days culture with medium alone without OES at 20% O_2_.

**Declarations**

**Ethics approval and consent to participate**

Not applicable

**Consent for publication**

Not applicable

**Availability of data and materials**

The RNA-seq datasets generated during the current study are available in the NCBI’s Sequence Read Archive (SRA), <http://www.ncbi.nlm.nih.gov/bioproject/1123936>, under the BioProject accession ID PRJNA1123936.

**Competing interests**

The authors declare that they have no competing interests.

**Funding**

This work was supported by the “Agence Nationale pour la Recherche” (Grant ORGALOG no. ANR-21-CE20-0042-01) and the Swiss National Foundation (Lead Agency project ORGALOG no. 205507). TP and RP were supported by The Royal Golden Jubilee (RGJ) Ph.D. Scholarship (PHD/0032/2560, 2.B.TS/60/F.1), Thailand Research Fund (TRF).

**Authors’ contributions**

PM, MSD, CA, and SB designed the study; TP, MSD, and SB wrote the manuscript; TP performed the co-cultures and analyzed the blastocysts with the help of CM and VLB; SB and CA performed sample processing and QC for RNA-seq and data analysis; ALV did the qRT-PCR analysis; RP participated in the analysis and interpretation of data; SB supervised the RNA analyses; all authors corrected the manuscript; MSD and SB administrated and supervised the study.

**Acknowledgements**

The authors are grateful to Albert Arnould for sample collection at the slaughterhouse, Marie-Claire Blache (Plateforme d’Imagerie Cellulaire, PRC Unit) for her help in confocal microscope and image analysis. The authors sincerely thank Synetics (France) for providing the bull semen. Furthermore, we would like to acknowledge the Functional Genomics Center Zurich (FGCZ) for performing RNA-seq library preparation and Illumina sequencing.

**FIGURE LEGENDS**

**Fig. 1. Experimental design of the study**.

Isthmic mucosa fragments (IMF) collected at slaughterhouse from cyclic cows near ovulation time were used to produce oviduct epithelial spheroids (OES). For co-culture with embryos, OES of 100-200 µm diameter with ciliary beating were selected. Eighteen hours after *in vitro* fertilization (IVF, Day 0), groups of 25 presumptive zygotes were co-cultured or not (controls=C) with 25 OES for 4 (4dOES) or 7 days (7dOES) and under low (5%) or high (20%) oxygen tension in 25-µl droplets of synthetic oviductal fluid (SOF) supplemented with 5% fetal bovine serum (FBS) under mineral oil. Cleavage and blastocyst rates were assessed on Days 2 and 7-8, respectively. On Days 7-8, expanded blastocysts were assessed for total cell number after fixation/nuclei staining, or frozen for gene expression analysis by RNA-sequencing.

**Fig. 2. Effect of co-culture with OES on cleavage, blastocyst rates and total cell numbers.** A, under 5% O_2_ level. Different letters indicate significant differences (P<0.05; N= 507-657 COCs per condition for cleavage and blastocysts rates; N=20 blastocysts per condition for cell numbers). B, under 20% O_2_ level. Different letters indicate significant differences (P<0.01; N= 768-795 COCs per condition for cleavage and blastocysts rates; N=20 blastocysts per condition for cell numbers).

**Fig. 3. Venn diagrams showing the overlaps of differentially expressed genes between different group comparisons. A** DEGs (FDR 1%) of the four comparisons of expanded blastocysts co-cultured with OES for 4 (4dOES) or 7 days (7dOES) to controls (C) without OES under 5% and 20% oxygen were compared. **B** Venn diagram comparing 7dOES-4dOES at 5% and 7dOES-4dOES at 20% O_2_. **C** Comparison of 5% vs. 20% O2 for 7dOES, 4dOES, and controls (C).

**Fig. 4. Heatmap of differentially expressed genes (DEGs) identified in expanded blastocysts according to co-culture conditions.** Expanded blastocysts were co-cultured or not (controls=C) with OES for 4 (4dOES) or 7 days (7dOES) and under high (20%) or low (5%) oxygen tension. Each row represents one DEG (FDR 1%) while each column represents one pool of blastocysts (6 pools/condition). Mean-centered log2-transformed counts per million values were obtained (value of respective sample – mean of all samples) for DEGs across experimental groups. Hierarchical cluster analysis was carried out using Pearson correlation (MeV software (39)). The blue color indicates lower expression than the mean of all samples whereas the red color indicates higher expression. At the top, the 6 different culture conditions are indicated.

**Fig. 5. Heatmap of differentially expressed genes (DEGs) obtained for the comparison between 4 and 7 days of co-culture groups.** Expanded blastocysts were co-cultured with OES for 4 (4dOES) or 7 days (7dOES) and under high (20%) or low (5%) oxygen tension. Each row represents one DEG (FDR 10%) while each column represents one pool of blastocysts (6 pools/condition). Mean-centered log2-transformed counts per million values were obtained (value of respective sample – mean of all samples) for DEGs across experimental groups. Hierarchical cluster analysis was carried out using Pearson correlation (MeV software (39)). The blue color indicates lower expression than the mean of all samples whereas the red color indicates higher expression. At the top, the 4 different culture conditions are indicated.

**Fig. 6. Heatmap of differentially expressed genes (DEGs) obtained for the comparison between 20% and 5% oxygen conditions.** Expanded blastocysts were co-cultured or not (controls=C) with OES for 4 (4dOES) or 7 days (7dOES) and under high (20%) or low (5%) oxygen tension. Each row represents one DEG (FDR 5%) while each column represents one pool of blastocysts (6 pools/condition). Mean-centered log2-transformed counts per million values were obtained (value of respective sample – mean of all samples) for DEGs across experimental groups. Hierarchical cluster analysis was carried out using Pearson correlation (MeV software (39)). The blue color indicates lower expression than the mean of all samples whereas the red color indicates higher expression. At the top, the 6 different culture conditions are indicated.

**Fig. 7. Functional annotation analysis of DEGs obtained for the comparison of embryos co-cultured with OES vs. controls.** Metascape analysis (40) was performed for the DEGs (FDR 1%) obtained for blastocysts co-cultured with OES for 4 (4dOES) or 7 days (7dOES) compared to controls (C) without OES in each oxygen group (5% and 20%; 4 comparisons). The Metascape heatmap shows the top 100 of the enriched clusters of functional terms. Each row indicates one functional term. Color indicates significance from orange-brown. Gray color indicates lack of significance. 7dOES–C 20%: co-culture with OES for 7 days vs. controls at 20% O_2_; 7dOES–C 5%: co-culture with OES for 7 days vs. controls at 5% O_2_; 4dOES–20%: co-culture with OES for 4 days vs. controls at 20% O_2_; 4dOES–C 5%: co-culture with OES for 4 days vs. controls at 5% O_2_.

**Fig. 8. Network of related functional terms for the DEGs obtained for the comparison of embryos co-cultured with OES vs. controls.** ToppCluster analysis (41) was performed for the DEGs (FDR 1%) obtained for blastocysts co-cultured with OES for 4 (4dOES) or 7 days (7dOES) compared to controls (C) without OES under 5% and 20% O_2_ (4 comparisons). Nodes in dark blue: GO Molecular functions, blue-green: GO cellular components, yellow: GO Biological processes, green: Pathways, brown: gene family, and light-yellow: enriched transcription factor binding sites. The thickness of the lines indicates significance. 4dOES_C_20: co-culture with OES for 4 days vs. controls at 20% O_2_; 4dOES_C_5: co-culture with OES for 4 days vs. controls at 5% O_2_; 7dOES_C_5: co-culture with OES for 7 days vs. controls at 5% O_2_; 7dOES_C_20: co-culture with OES for 7 days vs. controls at 20% O_2_.

**Fig. 9. Functional annotation analysis of DEGs obtained for the comparison between 20% and 5% oxygen conditions.** Metascape analysis (40) was performed for the DEGs (FDR 5%) obtained for blastocysts cultured under 5% O_2_ vs. 20% O_2_ for each culture group (4 days with OES; 7 days with OES; and controls without OES). The Metascape heatmap shows the top 100 of the enriched clusters of functional terms. Each row indicates one functional term. Color indicates significance from orange-brown. Gray color indicates lack of significance. 7dOES 20 vs. 5%: co-culture with OES for 7 days at 20% O_2_ vs. 5% O_2_; 4dOES 20 vs. 5%: co-culture with OES for 4 days at 20% O_2_ vs. 5% O_2_; C 20% vs. 5%: culture without OES for 7 days at 20% O_2_ vs. 5% O_2_.

**TABLES**

**Table 1.** Numbers of differentially expressed genes (DEGs) obtained for the different comparisons.

| Effect | Comparison | FDR 1% | | | | FDR 5% | | | | FDR 10% | | |
| --- | --- | --- | --- | --- | --- | --- | --- | --- | --- | --- | --- | --- |
|  |  | **total** | **up** | **down** | **total** | | **up** | **down** | **total** | | **up** | **down** |
| 4 days co-culture | 4dOES_5 vs. C_5 | 568 | 286 | 282 | 1255 | | 630 | 625 |  | |  |  |
| 7 days co-culture | 7dOES_5 vs. C_5 | 911 | 458 | 453 | 1874 | | 949 | 925 |  | |  |  |
| 4 days co-culture | 4dOES_20 vs. C_20 | 559 | 308 | 251 | 1457 | | 689 | 768 |  | |  |  |
| 7 days co-culture | 7dOES_20 vs. C_20 | 1282 | 691 | 591 | 2361 | | 1121 | 1240 |  | |  |  |
| Time of co-culture | 7dOES_5 vs. 4dOES_5 | 2 | 0 | 2 | 2 | | 0 | 2 | 2 | | 0 | 2 |
| Time of co-culture | 7dOES_20 vs. 4dOES_20 | 8 | 3 | 5 | 56 | | 29 | 27 | 292 | | 127 | 165 |
| % O_2_ | C_20 vs. C_5 | 238 | 169 | 69 | 767 | | 268 | 499 |  | |  |  |
| % O_2_ | 4dOES_20 vs. 4dOES_5 | 278 | 153 | 125 | 850 | | 411 | 439 |  | |  |  |
| % O_2_ | 7dOES_20 vs. 7dOES_5 | 45 | 32 | 13 | 127 | | 62 | 65 |  | |  |  |

Differential gene expression analysis was performed according to the presence of OES, time of co-culture, oxygen tension (effect). FDR: false discovery rate; up: number of DEGs with higher expression levels in first group of contrast (e.g., for 4dOES_5 vs. C_5 higher in 4dOES_5).

**Table 2.** Validation of RNA-seq results by quantitative real-time RT-PCR for selected genes

| Bta Gene ID | Bta symbol | Method | 4dOES–C 5% | FDR/  P-value | 4dOES–C 20% | FDR/  P-value | 7dOES–C 5% | FDR/  P-value | 7dOES–C 20% | FDR/  P-value |
| --- | --- | --- | --- | --- | --- | --- | --- | --- | --- | --- |
| 504615 | *FETUB* | RNA-seq | -1.43 | 0.000 | -1.32 | 0.000 | -1.39 | 0.000 | -1.73 | 0.000 |
|  |  | qPCR | -0.98 | 0.148 | -0.52 | 0.300 | -0.84 | 0.174 | -1.16 | 0.012 |
| 540085 | *GAB1* | RNA-seq | 0.78 | 0.000 | 0.68 | 0.000 | 0.90 | 0.000 | 1.04 | 0.000 |
|  |  | qPCR | 1.99 | 0.011 | 0.90 | 0.066 | 0.73 | 0.538 | 0.51 | 0.296 |
| 509200 | *HNF4A* | RNA-seq | 0.40 | 0.306 | 0.96 | 0.007 | 0.51 | 0.150 | 1.03 | 0.001 |
|  |  | qPCR | 0.78 | 0.142 | na | na | 0.74 | 0.132 | 1.61 | 0.014 |
| 281824 | *HSD3B1* | RNA-seq | 1.58 | 0.000 | 1.61 | 0.000 | 1.58 | 0.000 | 1.86 | 0.000 |
|  |  | qPCR | 1.76 | 0.003 | 1.25 | 0.094 | 1.60 | 0.018 | 1.30 | 0.045 |
| 100295610 | *MUC13* | RNA-seq | 1.46 | 0.000 | 1.36 | 0.001 | 2.09 | 0.000 | 1.26 | 0.001 |
|  |  | qPCR | na | na | 1.35 | 0.111 | 3.47 | 0.001 | 2.34 | 0.020 |
| 614673 | *NUPR1* | RNA-seq | 0.98 | 0.000 | 0.97 | 0.000 | 1.12 | 0.000 | 1.07 | 0.000 |
|  |  | qPCR | 1.34 | 0.006 | 1.34 | 0.000 | 1.21 | 0.005 | 1.13 | 0.005 |
| 505849 | *TFAP2A* | RNA-seq | 0.61 | 0.069 | 0.73 | 0.028 | 0.66 | 0.031 | 0.94 | 0.002 |
|  |  | qPCR | 2.10 | 0.032 | 1.56 | 0.020 | 1.05 | 0.213 | 0.76 | 0.186 |
| 514394 | *UCHL1* | RNA-seq | -1.08 | 0.055 | -0.05 | 0.957 | -1.88 | 0.000 | -2.06 | 0.000 |
|  |  | qPCR | -1.33 | 0.019 | -0.41 | 0.266 | -1.25 | 0.053 | -1.42 | 0.032 |
| 522980 | *USP2* | RNA-seq | 0.77 | 0.000 | 0.63 | 0.003 | 0.86 | 0.000 | 0.72 | 0.000 |
|  |  | qPCR | 0.79 | 0.277 | 2.40 | 0.054 | 0.98 | 0.025 | 0.92 | 0.159 |
|  |  |  | 20%–5% 4dOES | FDR/  P-value | 20%–5% 7dOES | FDR/  P-value | 20%–5% C | FDR/  P-value |  |  |
| 504615 | *FETUB* | RNA-seq | 1.00 | 0.032 | 0.55 | 0.450 | 0.89 | 0.029 |  |  |
|  |  | qPCR | 1.55 | 0.042 | 0.77 | 0.185 | 1.09 | 0.034 |  |  |
| 540085 | *GAB1* | RNA-seq | -0.51 | 0.008 | -0.27 | 0.377 | -0.41 | 0.066 |  |  |
|  |  | qPCR | -1.66 | 0.027 | -0.80 | 0.507 | -0.58 | 0.172 |  |  |
| 509200 | *HNF4A* | RNA-seq | -0.29 | 0.535 | -0.34 | 0.598 | -0.86 | 0.024 |  |  |
|  |  | qPCR | na | na | -0.76 | 0.185 | -1.64 | 0.009 |  |  |
| 614673 | *NUPR1* | RNA-seq | -0.50 | 0.010 | -0.53 | 0.022 | -0.48 | 0.017 |  |  |
|  |  | qPCR | -0.42 | 0.244 | -0.49 | 0.162 | -0.42 | 0.167 |  |  |
| 514394 | *UCHL1* | RNA-seq | 1.43 | 0.011 | 0.22 | 0.890 | 0.40 | 0.635 |  |  |
|  |  | qPCR | 0.82 | 0.121 | -0.26 | 0.695 | -0.09 | 0.778 |  |  |
|  |  |  | 7-4dOES 5% | FDR/  P-value | 7-4dOES 20% | FDR/  P-value |  |  |  |  |
| 514394 | *UCHL1* | RNA-seq | -0.80 | 0.998 | -2.01 | 0.005 |  |  |  |  |
|  |  | qPCR | 0.07 | 0.907 | -1.01 | 0.106 |  |  |  |  |

**SUPPLEMENTARY DATA**

**Supplementary video 1.** Oviduct epithelial spheroids after selection for co-culture with presumptive zygotes. The movements are due to the beating of ciliated cells oriented outward.

**Supplementary figure 1.** Representative images of blastocysts after nuclei staining**.**

A-C, Blastocysts cultured under 5% oxygen (O_2_) alone (A) or with oviduct epithelial spheroids (OES) for 4 days (B) or 7 days (C). D-F, Blastocysts cultured under 20% oxygen (O_2_) alone (D) or with oviduct epithelial spheroids (OES) for 4 days (E) or 7 days (F).

**Supplementary data 1.** Lists of differentially expressed genes (DEGs) and RNA-seq data obtained for the effects of OES co-culture (sheet 1), time of co-culture (sheet 2); and oxygen level (sheet 3).

**Supplementary data 2.** Lists of significant functional terms and Metascape analysis data for the effects of OES co-culture (sheet 1), time of co-culture (sheet 2); and oxygen level (sheet 3).

**Table S1. Primer sequences for quantitative real-time RT-PCR.**

| Primer | Sequence (5'->3') | Length | Product size | Gene ID | Gene symbol | Gene name | Acc. No. |
| --- | --- | --- | --- | --- | --- | --- | --- |
| FETUB_for | CATGTGTCAAGTCGGAGGGT | 20 | 166 | 504615 | *FETUB* | fetuin B | NM_001034218.2 |
| FETUB_rev | TTTTCACCTCTAGGCGTGGG | 20 | 166 | 504615 | *FETUB* | fetuin B | NM_001034218.2 |
| GAB1_for | CCCGATGATCAAGCCCAAAG | 20 | 168 | 540085 | *GAB1* | GRB2 associated binding protein 1 | NM_001101201.1 |
| GAB1_rev | AGAGCGAGGGTCTTCTGTTG | 20 | 168 | 540085 | *GAB1* | GRB2 associated binding protein 1 | NM_001101201.1 |
| HNF4A_for | GCGCATCCTTGATGAGTTGG | 20 | 182 | 509200 | *HNF4A* | hepatocyte nuclear factor 4 alpha | NM_001015557.1 |
| HNF4A_rev | GGCGGTCGTTGATGTAGTCT | 20 | 182 | 509200 | *HNF4A* | hepatocyte nuclear factor 4 alpha | NM_001015557.1 |
| HSD3B1_for | GACGTCAGGAATGCTGTCCC | 20 | 129 | 281824 | *HSD3B1* | hydroxy-delta-5-steroid dehydrogenase, 3 beta- and steroid delta-isomerase 1 | NM_174343.3 |
| HSD3B1_rev | AGCCACTTCTATGGTGCTGG | 20 | 129 | 281824 | *HSD3B1* | hydroxy-delta-5-steroid dehydrogenase, 3 beta- and steroid delta-isomerase 1 | NM_174343.3 |
| MUC13_for | GAACCCCTACACAAGGCACA | 20 | 143 | 100295610 | *MUC13* | mucin 13, cell surface associated | XM_002684796.7 |
| MUC13_rev | AGATCTTCAAGGCTGCTCCC | 20 | 143 | 100295610 | *MUC13* | mucin 13, cell surface associated | XM_002684796.7 |
| NUPR1_for | GACCAAGCTTCAGAACACGG | 20 | 189 | 614673 | *NUPR1* | nuclear protein 1, transcriptional regulator | NM_001114515.2 |
| NUPR1_rev | AGTCTCAGTCAGGTGGGGTTA | 21 | 189 | 614673 | *NUPR1* | nuclear protein 1, transcriptional regulator | NM_001114515.2 |
| TFAP2A_for | ACATTCCGATCCCAATGAGCA | 21 | 80 | 505849 | *TFAP2A* | transcription factor AP-2 alpha | XM_010818584.4 |
| TFAP2A_rev | GGTCGGTGAACTCCTTGCAG | 20 | 80 | 505849 | *TFAP2A* | transcription factor AP-2 alpha | XM_010818584.4 |
| UCHL1_for | GTGGATGGCCACCTCTATGA | 20 | 173 | 514394 | *UCHL1* | ubiquitin C-terminal hydrolase L1 | NM_001046172.2 |
| UCHL1_rev | CATTAGGCCGCCTTGCAGA | 19 | 173 | 514394 | *UCHL1* | ubiquitin C-terminal hydrolase L1 | NM_001046172.2 |
| USP2_for | GCTGGTCTTCGAAACCTTGG | 20 | 91 | 522980 | *USP2* | ubiquitin specific peptidase 2 | NM_001046263.1 |
| USP2_rev | GCAGGCAGTAGTCTCTCAGC | 20 | 91 | 522980 | *USP2* | ubiquitin specific peptidase 2 | NM_001046263.1 |
| ACTB_f | CCGCAACCAGTTCGCCATGGATG | 23 | 192 | 280979 | *ACTB* | actin beta | NM_173979.3 |
| ACTB_r | TGAGCCTCATCCCCCACGTACG | 22 | 192 | 280979 | *ACTB* | actin beta | NM_173979.3 |
| GAPDH _f | GTCGGAGTGAACGGATTCGG | 20 | 193 | 281181 | *GAPDH* | glyceraldehyde-3-phosphate dehydrogenase | NM_001034034.2 |
| GAPDH _r | TGATGACGAGCTTCCCGTTC | 20 | 193 | 281181 | *GAPDH* | glyceraldehyde-3-phosphate dehydrogenase | NM_001034034.2 |
| H3F3A_f | CTGCCCGCAAATCGACCGGT | 20 | 199 | 326601 | *H3-3A* | H3.3 histone A | NM_001014389.2 |
| H3F3A_r | CGCACCAGACGCTGGAAGGG | 20 | 199 | 326601 | *H3-3A* | H3.3 histone A | NM_001014389.2 |
| UBB_f | CGTCTGAGGGGTGGCTGTTA | 20 | 84 | 281370 | *UBB* | ubiquitin B | NM_174133.2 |
| UBB_r | GGGTAAATGGCTAGAGTGCAGA | 22 | 84 | 281370 | *UBB* | ubiquitin B | NM_174133.2 |

**Table S2.** Effect of co-culture with or without OES under 5% and 20% O_2_ on blastocyst yield and cell number.

| Time of co-culture | N | % Cleavage  Rate (n) | % Blastocyst rate (n)  Day 7 | %Blastocyst rate (n)  Day 8 | Cell number per expanded blastocyst (n) |  |
| --- | --- | --- | --- | --- | --- | --- |
| Under low O_2_ level (5%) | | | | | | |
| Control | 557 | 78.4 ± 3.2 (444) | 26.1 ± 3.9 (150) | 30.4 ± 4.5 (177) | 102.6 ± 8.4^a^ (20) |  |
| 4 days | 657 | 78 ± 3.3 (514) | 20.8 ± 3.1 (139) | 27.3 ± 3 (181) | 112.9 ± 6.7^ab^ (20) |  |
| 7 days | 507 | 81.4 ± 3.2 (417) | 23.9 ± 3.1 (117) | 31.8 ± 3 (155) | 137.6 ± 10.8^b^ (20) |  |
| Under high O_2_ level (20%) | | | | | | |
| Control | 790 | 72.8 ± 2.9 (580) | 17 ± 1.9^a^ (132) | 19.8 ± 2.4^a^ (151) | 82.1 ± 4.5^a^ (20) |  |
| 4 days | 768 | 78.6 ± 2 (604) | 27.6 ± 2.2^b^ (215) | 30.7 ± 2^b^ (236) | 112.7 ± 7.8^b^ (20) |  |
| 7 days | 795 | 78.9 ± 2 (623) | 26 ± 2.3^b^ (208) | 31.8 ± 2.5^b^ (248) | 138.1 ± 10.5^b^ (20) |  |

Percentage values are provided as the means ± SEM of 8-9 replicates except blastocyst cell numbers (3 replicates). Different letters within one column indicate significant differences (P<0.05)^a,b^. N = total number of cumulus-oocyte complexes; and n = total number of embryos.

**Table S3.** Effect of oxygen tension in presence or absence of OES on blastocyst yield and cell number.

| Time of co-culture | N | % Cleavage  rate (n) | % Blastocyst rate (n)  Day 7 | %Blastocyst rate (n)  Day 8 | Cell number per expanded blastocyst (n) | |
| --- | --- | --- | --- | --- | --- | --- |
| In absence of OES (control) | | | | | |  |
| C_5% O_2_ | 557 | 78.4 ± 3.2 (444) | 26.1 ± 3.9 (150) | 30.4 ± 4.5^a^ (177) | 102.6 ± 8.4^x^ (20) | |
| C_20% O_2_ | 790 | 72.8 ± 2.9 (580) | 17 ± 1.9 (132) | 19.8 ± 2.4^b^ (151) | 82.1 ± 4.5^y^ (20) | |
| In the presence of OES for 4 days | | | | | |  |
| OES_4_5% | 657 | 78 ± 3.3 (514) | 20.8 ± 3.1 (139) | 27.3 ± 3 (181) | 112.9 ± 6.7 (20) | |
| OES_4_20% | 768 | 78.6 ± 2 (604) | 27.6 ± 2.2 (215) | 30.7 ± 2 (236) | 112.7 ± 7.8 (20) | |
| In the presence of OES for 7 days | | | | | |  |
| OES_7_5% | 507 | 81.4 ± 3.2 (417) | 23.9 ± 3.1 (117) | 31.8 ± 3 (155) | 137.6 ± 10.8 (20) | |
| OES_7_20% | 795 | 78.9 ± 2 (623) | 26 ± 2.3 (208) | 31.8 ± 2.5 (248) | 138.1 ± 10.5 (20) | |

Percentage values are provided as the means ± SEM of 8 replicates under 5% O_2_ and 9 replicates under 20% O_2_, except blastocyst cell numbers (3 replicates in both oxygen tensions). The rates of cleavage and blastocysts and the mean of cell number per expanded blastocyst were analyzed using unpaired t-test (P<0.05)^x,y^. N = total number of cumulus-oocyte complexes; and n = total number of embryos.

**References**

1. Joao H. M. Viana C. 2022 Statistics of embryo production and transfer in domestic farm animals. Embryo Technology Newsletter. 2023; v. 41, n.4.

2. Enright BP, Lonergan P, Dinnyes A, Fair T, Ward FA, Yang X, et al. Culture of in vitro produced bovine zygotes in vitro vs in vivo: implications for early embryo development and quality. Theriogenology. 2000;54(5):659-73.

3. Rizos D, Ward F, Duffy P, Boland MP, Lonergan P. Consequences of bovine oocyte maturation, fertilization or early embryo development in vitro versus in vivo: implications for blastocyst yield and blastocyst quality. Mol Reprod Dev. 2002;61(2):234-48.

4. Maillo V, Sanchez-Calabuig MJ, Lopera-Vasquez R, Hamdi M, Gutierrez-Adan A, Lonergan P, et al. Oviductal response to gametes and early embryos in mammals. Reproduction. 2016;152(4):R127-41.

5. Rodríguez-Alonso B, Sánchez JM, González E, Lonergan P, Rizos D. Challenges in studying preimplantation embryo-maternal interaction in cattle. Theriogenology. 2020;150:139-49.

6. Rizos D, Fair T, Papadopoulos S, Boland MP, Lonergan P. Developmental, qualitative, and ultrastructural differences between ovine and bovine embryos produced in vivo or in vitro. Mol Reprod Dev. 2002;62(3):320-7.

7. Abe H, Otoi T, Tachikawa S, Yamashita S, Satoh T, Hoshi H. Fine structure of bovine morulae and blastocysts in vivo and in vitro. Anat Embryol (Berl). 1999;199(6):519-27.

8. Crosier AE, Farin PW, Dykstra MJ, Alexander JE, Farin CE. Ultrastructural morphometry of bovine blastocysts produced in vivo or in vitro. Biol Reprod. 2001;64(5):1375-85.

9. Janati Idrissi S, Le Bourhis D, Lefevre A, Emond P, Le Berre L, Desnoës O, et al. Lipid profile of bovine grade-1 blastocysts produced either in vivo or in vitro before and after slow freezing process. Scientific Reports. 2021;11(1):11618.

10. Lonergan P, Rizos D, Gutierrez-Adan A, Moreira PM, Pintado B, de la Fuente J, et al. Temporal divergence in the pattern of messenger RNA expression in bovine embryos cultured from the zygote to blastocyst stage in vitro or in vivo. Biol Reprod. 2003;69(4):1424-31.

11. Driver AM, Peñagaricano F, Huang W, Ahmad KR, Hackbart KS, Wiltbank MC, et al. RNA-Seq analysis uncovers transcriptomic variations between morphologically similar in vivo- and in vitro-derived bovine blastocysts. BMC Genomics. 2012;13(1):118.

12. Gad A, Hoelker M, Besenfelder U, Havlicek V, Cinar U, Rings F, et al. Molecular mechanisms and pathways involved in bovine embryonic genome activation and their regulation by alternative in vivo and in vitro culture conditions. Biol Reprod. 2012;87(4):100.

13. Lange-Consiglio A, Lazzari B, Pizzi F, Idda A, Cremonesi F, Capra E. Amniotic microvesicles impact hatching and pregnancy percentages of in vitro bovine embryos and blastocyst microRNA expression versus in vivo controls. Scientific Reports. 2020;10(1):501.

14. Banliat C, Mahe C, Lavigne R, Com E, Pineau C, Labas V, et al. The proteomic analysis of bovine embryos developed in vivo or in vitro reveals the contribution of the maternal environment to early embryo. BMC Genomics. 2022;23(1):839.

15. Graf A, Krebs S, Heininen-Brown M, Zakhartchenko V, Blum H, Wolf E. Genome activation in bovine embryos: review of the literature and new insights from RNA sequencing experiments. Anim Reprod Sci. 2014;149(1-2):46-58.

16. Fischer B, Bavister BD. Oxygen tension in the oviduct and uterus of rhesus monkeys, hamsters and rabbits. J Reprod Fertil. 1993;99(2):673-9.

17. Yuan YQ, Van Soom A, Coopman FO, Mintiens K, Boerjan ML, Van Zeveren A, et al. Influence of oxygen tension on apoptosis and hatching in bovine embryos cultured in vitro. Theriogenology. 2003;59(7):1585-96.

18. Karagenc L, Sertkaya Z, Ciray N, Ulug U, Bahçeci M. Impact of oxygen concentration on embryonic development of mouse zygotes. Reproductive BioMedicine Online. 2004;9(4):409-17.

19. Leite RF, Annes K, Ispada J, de Lima CB, Dos Santos É C, Fontes PK, et al. Oxidative Stress Alters the Profile of Transcription Factors Related to Early Development on In Vitro Produced Embryos. Oxid Med Cell Longev. 2017;2017:1502489.

20. Yang Y, Xu Y, Ding C, Khoudja RY, Lin M, Awonuga AO, et al. Comparison of 2, 5, and 20 % O2 on the development of post-thaw human embryos. J Assist Reprod Genet. 2016;33(7):919-27.

21. Cordova A, Perreau C, Uzbekova S, Ponsart C, Locatelli Y, Mermillod P. Development rate and gene expression of IVP bovine embryos cocultured with bovine oviduct epithelial cells at early or late stage of preimplantation development. Theriogenology. 2014;81(9):1163-73.

22. Schmaltz-Panneau B, Locatelli Y, Uzbekova S, Perreau C, Mermillod P. Bovine Oviduct Epithelial Cells Dedifferentiate Partly in Culture, While Maintaining their Ability to Improve Early Embryo Development Rate and Quality. Reprod Domest Anim. 2015;50(5):719-29.

23. Clemente M, de la Fuente J, Lonergan P, Gutierrez-Adan A, Rizos D. Effect of Oxygen Tension on Embryo Development and Gene Transcription of Bovine Blastocysts Produced In Vitro by Co-culture with Oviduct Epithelial Cells. Biology of Reproduction. 2008;78(Suppl_1):133-.

24. Lopera-Vásquez R, Hamdi M, Fernandez-Fuertes B, Maillo V, Beltrán-Breña P, Calle A, et al. Extracellular Vesicles from BOEC in In Vitro Embryo Development and Quality. PLoS One. 2016;11(2):e0148083.

25. Alminana C, Corbin E, Tsikis G, Alcantara-Neto AS, Labas V, Reynaud K, et al. Oviduct extracellular vesicles protein content and their role during oviduct-embryo cross-talk. Reproduction. 2017;154(3):153-68.

26. Kürüm A, Karahan S, KocamiŞ H, Cinar M, Ergün E. Determination of antioxidants in bovine oviduct epithelial cell culture isolated at different periods of the estrous cycle. TURKISH JOURNAL OF VETERINARY AND ANIMAL SCIENCES. 2019;43:448-55.

27. Pedersen ME, Øzdas Ø B, Farstad W, Tverdal A, Olsaker I. Effects of bovine oviduct epithelial cells, fetal calf serum and bovine serum albumin on gene expression in single bovine embryos produced in the synthetic oviduct fluid culture system. Reprod Fertil Dev. 2005;17(8):751-7.

28. Pranomphon T, Mahé C, Demattei M-V, Papillier P, Vitorino Carvalho A, Reynaud K, et al. Characterization of oviduct epithelial spheroids for the study of embryo–maternal communication in cattle. Theriogenology. 2024;217:113-26.

29. Bauersachs S, Mermillod P, Alminana C. The Oviductal Extracellular Vesicles' RNA Cargo Regulates the Bovine Embryonic Transcriptome. Int J Mol Sci. 2020;21(4).

30. Mermillod P, Vansteenbrugge A, Wils C, Mourmeaux JL, Massip A, Dessy F. Characterization of the embryotrophic activity of exogenous protein-free oviduct-conditioned medium used in culture of cattle embryos. Biol Reprod. 1993;49(3):582-7.

31. Donnay I, Faerge I, Grøndahl C, Verhaeghe B, Sayoud H, Ponderato N, et al. Effect of prematuration, meiosis activating sterol and enriched maturation medium on the nuclear maturation and competence to development of calf oocytes. Theriogenology. 2004;62(6):1093-107.

32. Lamy J, Corbin E, Blache MC, Garanina AS, Uzbekov R, Mermillod P, et al. Steroid hormones regulate sperm-oviduct interactions in the bovine. Reproduction. 2017;154(4):497-508.

33. Parrish JJ, Susko-Parrish J, Winer MA, First NL. Capacitation of bovine sperm by heparin. Biol Reprod. 1988;38(5):1171-80.

34. Holm P, Booth PJ, Schmidt MH, Greve T, Callesen H. High bovine blastocyst development in a static in vitro production system using SOFaa medium supplemented with sodium citrate and myo-inositol with or without serum-proteins. Theriogenology. 1999;52(4):683-700.

35. Mahé C, Pranomphon T, Reynaud K, Laffont L, Meylheuc T, Schoen J, et al. Sperm-fluid-cell interplays in the bovine oviduct: glycosaminoglycans modulate sperm binding to the isthmic reservoir. Scientific Reports. 2023;13(1):10311.

36. The Galaxy platform for accessible, reproducible and collaborative biomedical analyses: 2022 update. Nucleic Acids Res. 2022;50(W1):W345-w51.

37. Robinson MD, McCarthy DJ, Smyth GK. edgeR: a Bioconductor package for differential expression analysis of digital gene expression data. Bioinformatics. 2010;26(1):139-40.

38. Hackstadt AJ, Hess AM. Filtering for increased power for microarray data analysis. BMC Bioinformatics. 2009;10:11.

39. Saeed AI, Sharov V, White J, Li J, Liang W, Bhagabati N, et al. TM4: a free, open-source system for microarray data management and analysis. Biotechniques. 2003;34(2):374-8.

40. Zhou Y, Zhou B, Pache L, Chang M, Khodabakhshi AH, Tanaseichuk O, et al. Metascape provides a biologist-oriented resource for the analysis of systems-level datasets. Nat Commun. 2019;10(1):1523.

41. Kaimal V, Bardes EE, Tabar SC, Jegga AG, Aronow BJ. ToppCluster: a multiple gene list feature analyzer for comparative enrichment clustering and network-based dissection of biological systems. Nucleic Acids Res. 2010;38(Web Server issue):W96-102.

42. Livak KJ, Schmittgen TD. Analysis of relative gene expression data using real-time quantitative PCR and the 2(-Delta Delta C(T)) Method. Methods. 2001;25(4):402-8.

43. R Core Team. R: A Language and Environment for Statistical Computing. R Foundation for Statistical Computing, Vienna, Austria. 2024. <https://www.R-project.org/>.

44. Lapointe J, Bilodeau JF. Antioxidant defenses are modulated in the cow oviduct during the estrous cycle. Biol Reprod. 2003;68(4):1157-64.

45. Banliat C, Tsikis G, Labas V, Teixeira-Gomes AP, Com E, Lavigne R, et al. Identification of 56 Proteins Involved in Embryo-Maternal Interactions in the Bovine Oviduct. Int J Mol Sci. 2020;21(2).

46. Harvey MB, Arcellana-Panlilio MY, Zhang X, Schultz GA, Watson AJ. Expression of genes encoding antioxidant enzymes in preimplantation mouse and cow embryos and primary bovine oviduct cultures employed for embryo coculture. Biol Reprod. 1995;53(3):532-40.

47. Fang X, Tanga BM, Bang S, Seong G, Saadeldin IM, Lee S, et al. Oviduct epithelial cells-derived extracellular vesicles improve preimplantation developmental competence of in vitro produced porcine parthenogenetic and cloned embryos. Mol Reprod Dev. 2022;89(1):54-65.

48. Ji HW, Wang CR, Yuan XW, Wang J, Wang L, Cao QL, et al. Mangiferin improves early porcine embryonic development by reducing oxidative stress. Reprod Domest Anim. 2024;59(4):e14565.

49. Prawan A, Buranrat B, Kukongviriyapan U, Sripa B, Kukongviriyapan V. Inflammatory cytokines suppress NAD(P)H:quinone oxidoreductase-1 and induce oxidative stress in cholangiocarcinoma cells. J Cancer Res Clin Oncol. 2009;135(4):515-22.

50. Hoelker M, Schmoll F, Schneider H, Rings F, Gilles M, Tesfaye D, et al. Bovine blastocyst diameter as a morphological tool to predict embryo cell counts, embryo sex, hatching ability and developmental characteristics after transfer to recipients. Reprod Fertil Dev. 2006;18(5):551-7.

51. Ross PJ, Goissis MD, Martins JPN, Chitwood JL, Pursley JR, Rosa GJM, et al. Blastocyst Cell Number and Allocation Affect the Developmental Potential and Transcriptome of Bovine Somatic Cell Nuclear Transfer Embryos. Stem Cells Dev. 2023;32(17-18):515-23.

52. Lopera-Vasquez R, Hamdi M, Maillo V, Lloreda V, Coy P, Gutierrez-Adan A, et al. Effect of bovine oviductal fluid on development and quality of bovine embryos produced in vitro. Reprod Fertil Dev. 2017;29(3):621-9.

53. Tse PK, Lee YL, Chow WN, Luk JM, Lee KF, Yeung WS. Preimplantation embryos cooperate with oviductal cells to produce embryotrophic inactivated complement-3b. Endocrinology. 2008;149(3):1268-76.

54. Edwards LJ, Batt PA, Gandolfi F, Gardner DK. Modifications made to culture medium by bovine oviduct epithelial cells: changes to carbohydrates stimulate bovine embryo development. Mol Reprod Dev. 1997;46(2):146-54.

55. Timme TL, Goltsov A, Tahir S, Li L, Wang J, Ren C, et al. Caveolin-1 is regulated by c-myc and suppresses c-myc-induced apoptosis. Oncogene. 2000;19(29):3256-65.

56. Santofimia-Castano P, Huang C, Liu X, Xia Y, Audebert S, Camoin L, et al. NUPR1 protects against hyperPARylation-dependent cell death. Commun Biol. 2022;5(1):732.

57. Wei Q, Zhong L, Zhang S, Mu H, Xiang J, Yue L, et al. Bovine lineage specification revealed by single-cell gene expression analysis from zygote to blastocyst. Biol Reprod. 2017;97(1):5-17.

58. Li G, Zhao CY, Wu Q, Kang Z, Zhang JT, Guan SY, et al. Di(2-ethylhexyl) phthalate disturbs cholesterol metabolism through oxidative stress in rat liver. Environ Toxicol Pharmacol. 2022;95:103958.

59. Van Winkle LJ. Amino acid transport regulation and early embryo development. Biol Reprod. 2001;64(1):1-12.

60. Tan BSN, Rathjen PD, Harvey AJ, Gardner DK, Rathjen J. Regulation of amino acid transporters in pluripotent cell populations in the embryo and in culture; novel roles for sodium-coupled neutral amino acid transporters. Mech Dev. 2016;141:32-9.

61. Hardy MLM, Lakhiani D, Morris MB, Day ML. Proline and Proline Analogues Improve Development of Mouse Preimplantation Embryos by Protecting Them against Oxidative Stress. Cells. 2023;12(22).

62. de Andrade Melo-Sterza F, Poehland R. Lipid Metabolism in Bovine Oocytes and Early Embryos under In Vivo, In Vitro, and Stress Conditions. International Journal of Molecular Sciences. 2021;22(7):3421.

63. Asaadi A, Kafi M, Atashi H, Azari M, Hostens M. Frozen-thawed ampullary cell monolayer improves bovine embryo in vitro development and quality. Zygote. 2019;27(5):337-46.

64. Lopera-Vasquez R, Hamdi M, Maillo V, Gutierrez-Adan A, Bermejo-Alvarez P, Ramírez M, et al. Effect of bovine oviductal extracellular vesicles on embryo development and quality in vitro. Reproduction. 2017;153(4):461-70.

65. Boruszewska D, Grycmacher K, Kowalczyk-Zieba I, Sinderewicz E, Staszkiewicz-Chodor J, Woclawek-Potocka I. Expression of enzymes involved in the synthesis of prostaglandin E(2) in early- and late-cleaved bovine embryos at different stages of preimplantation development. Theriogenology. 2019;133:45-55.

66. Saint-Dizier M, Guyader-Joly C, Charpigny G, Grimard B, Humblot P, Ponter AA. Expression of enzymes involved in the synthesis of prostaglandin E2 in bovine in vitro-produced embryos. Zygote. 2011;19(3):277-83.

67. Miller WL, Auchus RJ. The molecular biology, biochemistry, and physiology of human steroidogenesis and its disorders. Endocr Rev. 2011;32(1):81-151.

68. Furbass R, Michaelis M, Schuler G. Unhatched bovine blastocysts express all transcripts of the estrogen biosynthetic pathway, but steroid hormone synthesis could not yet be demonstrated. Domest Anim Endocrinol. 2023;82:106770.

69. Schmokel V, Memar N, Wiekenberg A, Trotzmuller M, Schnabel R, Doring F. Genetics of Lipid-Storage Management in Caenorhabditis elegans Embryos. Genetics. 2016;202(3):1071-83.

70. Lee SY, Hong IK, Kim BR, Shim SM, Sung Lee J, Lee HY, et al. Activation of sphingosine kinase 2 by endoplasmic reticulum stress ameliorates hepatic steatosis and insulin resistance in mice. Hepatology. 2015;62(1):135-46.

71. Glajch KE, Moors TE, Chen Y, Bechade PA, Nam AY, Rajsombath MM, et al. Wild-type GBA1 increases the alpha-synuclein tetramer-monomer ratio, reduces lipid-rich aggregates, and attenuates motor and cognitive deficits in mice. Proc Natl Acad Sci U S A. 2021;118(31).

72. Lee MT, Bonneau AR, Giraldez AJ. Zygotic genome activation during the maternal-to-zygotic transition. Annu Rev Cell Dev Biol. 2014;30:581-613.

73. Badr H, Bongioni G, Abdoon AS, Kandil O, Puglisi R. Gene expression in the in vitro-produced preimplantation bovine embryos. Zygote. 2007;15(4):355-67.

74. Pohler KG, Franco GA, Reese ST, Smith MF. Chapter 3 - Physiology and pregnancy of beef cattle. In: Bazer FW, Lamb GC, Wu G, editors. Animal Agriculture: Academic Press; 2020. p. 37-55.

75. Dalvit GC, Cetica PD, Pintos LN, Beconi MT. Reactive oxygen species in bovine embryo in vitro production. Biocell. 2005;29(2):209-12.

76. Lonergan P, Forde N. Maternal-embryo interaction leading up to the initiation of implantation of pregnancy in cattle. Animal. 2014;8 Suppl 1:64-9.

77. Ozawa M, Yang QE, Ealy AD. The expression of fibroblast growth factor receptors during early bovine conceptus development and pharmacological analysis of their actions on trophoblast growth in vitro. Reproduction. 2013;145(2):191-201.

78. Molotkov A, Mazot P, Brewer JR, Cinalli RM, Soriano P. Distinct Requirements for FGFR1 and FGFR2 in Primitive Endoderm Development and Exit from Pluripotency. Dev Cell. 2017;41(5):511-26 e4.

79. Ge D, Kong X, Liu W, Zhao J, Su L, Zhang S, et al. Phosphorylation and nuclear translocation of integrin beta4 induced by a chemical small molecule contribute to apoptosis in vascular endothelial cells. Apoptosis. 2013;18(9):1120-31.

80. Israel S, Seyfarth J, Nolte T, Drexler HCA, Fuellen G, Boiani M. Intracellular fraction of zona pellucida protein 3 is required for the oocyte-to-embryo transition in mice. Mol Hum Reprod. 2023;29(11).

81. Wan Y, Li D, Deng M, Liu Z, Liu L, Wang F. Comprehensive Transcriptome Analysis of mRNA Expression Patterns of Early Embryo Development in Goat under Hypoxic and Normoxic Conditions. Biology (Basel). 2021;10(5).

82. Sang Q, Zhou Z, Mu J, Wang L. Genetic factors as potential molecular markers of human oocyte and embryo quality. J Assist Reprod Genet. 2021;38(5):993-1002.

83. Jiao ZX, Woodruff TK. Detection and quantification of maternal-effect gene transcripts in mouse second polar bodies: potential markers of embryo developmental competence. Fertil Steril. 2013;99(7):2055-61.

84. Sperandio S, Fortin J, Sasik R, Robitaille L, Corbeil J, de Belle I. The transcription factor Egr1 regulates the HIF-1alpha gene during hypoxia. Mol Carcinog. 2009;48(1):38-44.

85. Tesfaye D, Regassa A, Rings F, Ghanem N, Phatsara C, Tholen E, et al. Suppression of the transcription factor MSX1 gene delays bovine preimplantation embryo development in vitro. Reproduction. 2010;139(5):857-70.

86. Gassler J, Kobayashi W, Gaspar I, Ruangroengkulrith S, Mohanan A, Gomez Hernandez L, et al. Zygotic genome activation by the totipotency pioneer factor Nr5a2. Science. 2022;378(6626):1305-15.

87. Jeyarajah MJ, Jaju Bhattad G, Kelly RD, Baines KJ, Jaremek A, Yang FP, et al. The multifaceted role of GCM1 during trophoblast differentiation in the human placenta. Proc Natl Acad Sci U S A. 2022;119(49):e2203071119.

88. Zhao XM, Cui LS, Hao HS, Wang HY, Zhao SJ, Du WH, et al. Transcriptome analyses of inner cell mass and trophectoderm cells isolated by magnetic-activated cell sorting from bovine blastocysts using single cell RNA-seq. Reprod Domest Anim. 2016;51(5):726-35.

89. Speckhart SL, Oliver MA, Keane JA, Dias NW, Mercadante VRG, Biase FH, et al. Interleukin-6 supplementation improves bovine conceptus elongation and transcriptomic indicators of developmental competence. Biol Reprod. 2024.

90. Wooldridge LK, Ealy AD. Interleukin-6 increases inner cell mass numbers in bovine embryos. BMC Dev Biol. 2019;19(1):2.
