# Supplementary figures and images for: Oviduct epithelial spheroids during in vitro culture of bovine embryos mitigate oxidative stress, improve blastocyst quality and change the embryonic transcriptome
Source: Biol Res. 2024 Oct 22;57:73. doi: 10.1186/s40659-024-00555-5 (PMC11494963; doi:10.1186/s40659-024-00555-5)

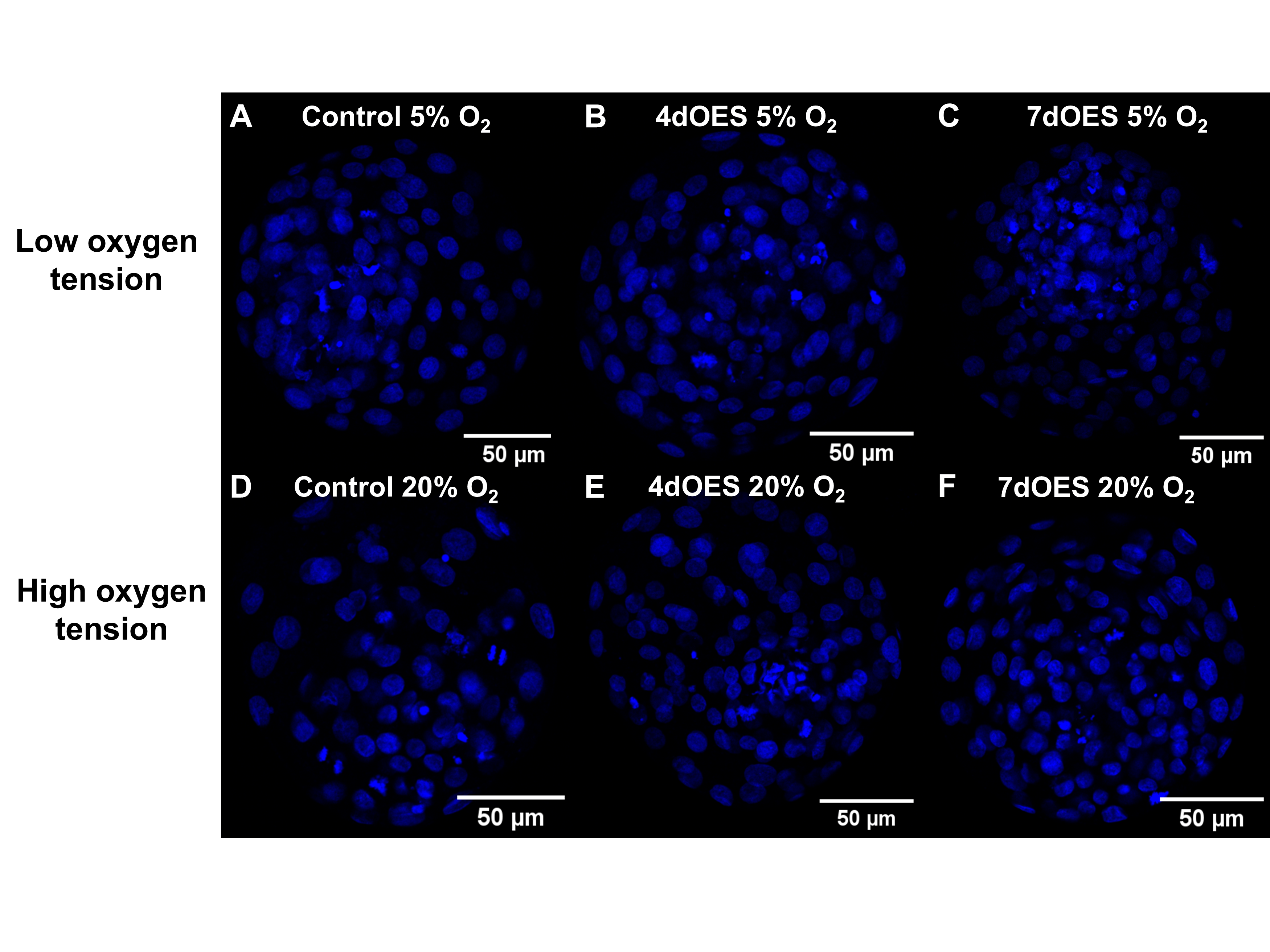

Supplement: Supplementary file 5 — Supplementary Material 5 [file 40659_2024_555_MOESM5_ESM.tif]
